# Dispersion interactions

**DOI:** 10.3762/bjoc.14.286

**Published:** 2018-12-18

**Authors:** Peter R Schreiner

**Affiliations:** 1 Institute of Organic Chemistry, Justus-Liebig University, Heinrich-Buff-Ring 17, 35392 Giessen, Germany

**Keywords:** London dispersion, van-der-Waals potential

**Figure 1:**
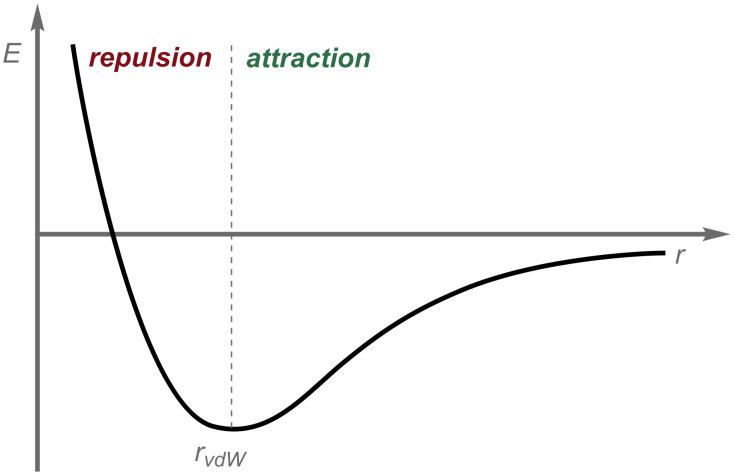
Dispersion = attractive part of the van-der-Waals potential.

London dispersion (LD) [1–3], the attractive part of the van-der-Waals [4] (vdW) potential (Figure 1), has long been recognized as an important binding interaction, just not so much in molecular organic, or for that matter, also inorganic chemistry. The repulsive part of the vdW potential has been well appreciated and is synonymous with the notion of “steric repulsion” [5–6]. The dilemma is that without a balanced description of attractive and repulsive forces, there cannot be a full understanding of structure and reactivity in chemistry. That is why we refer to “equilibrium structures” in spectroscopy and theory because there is perfect (time-averaged) equilibrium of all forces at work: they sum up to zero. The neglect of the attractive part of the vdW potential probably derives from the fact that there is no classic analogue as in the case of repulsion for which the hard sphere atom model works well.

LD is a purely quantum mechanical effect due to electron correlation. It is present for all matter starting from atoms (e.g., the condensation of helium) over molecules (e.g., aggregation) to materials (e.g., adhesion). It is also difficult to include in theoretical approaches and for the longest time it had been left virtually unnoticed that many density functional theory (DFT) approaches largely lacked the inclusion of LD. The often very good results of standard DFT implementations in the description of chemical reactions can in part be traced back to the compensating effects of neglecting both dispersion and solvation. This is not to say that dispersion vanishes [7] in solution but it is certainly attenuated [8]. We are still in the process of understanding just by how much.

LD is a driving force for molecular aggregation that plays a key role in the thermodynamic stability, molecular recognition, chemical selectivity through transition-state stabilization, protein folding, enzyme catalysis, and much more. Hence, this thematic issue covers selected aspects of the role LD plays for structures and reactivity. Naturally, it addresses diverse topics for which LD is particularly apparent.

Peter R. Schreiner

Giessen, November 2018
